# Supplementary material for: Virtual multi-alignment theory of parallel-beam CT image reconstruction for elastic objects
Source: Sci Rep. 2019 May 2;9:6847. doi: 10.1038/s41598-019-43331-2 (PMC6497627; doi:10.1038/s41598-019-43331-2)
Supplement: Supplementary file 1 — All supplementary files [file 41598_2019_43331_MOESM1_ESM.zip › Marked_Manuscript.pdf]

Virtual multi-alignment theory of parallel-beam CT image reconstruction for elastic objects

Kyungtaek Jun[a,*], Joeun Jung[b]

[a]IM Technology Research Center, 6, Teheran-ro 52-gil, Gangnam-gu, Seoul, 06211, Republic of Korea

[b]School of Mathematics, Korea Institute for Advanced Study, 85 Hoegiro, Dongdaemungu, Seoul, 02455, Republic of Korea

*ktfriends@gmail.com

Abstract

In parallel-beam tomography, the virtual alignment method plays an important role in obtaining an ideally aligned reconstruction of a rigid specimen. Furthermore, the method has been developed for an elastic specimen with specific motions such as periodic motion, regular expansion or contraction, and elliptical expansion or contraction to obtain a sinogram with an ideal sinogram pattern by transforming an elastic-type projection image set into a rigid-type projection image set satisfying the Helgason-Ludwig consistency condition. In this article, we present a method to convert a combined elastic specimen to a rigid specimen using the virtual multi-alignment method that allows us to obtain an ideally multi-aligned reconstruction of a combined elastic specimen.

Introduction

X-ray tomography has been a critical technique for structural studies in various fields ranging from biology to geosciences and materials science[1,2,3,4,5,6]. It is a mathematical technique for constructing 3D structures from a series of X-ray projection images at different angles of a fixed axis. Moreover, the technique has become significantly important as the only imaging technique that allows three-dimensional views of intracellular structure. However, since electron micrographs with high resolution for high-throughput experiments have been developed, problems have resulted from misalignment of the rotation axis of the series of projections. Subsequently, to obtain well-aligned reconstructed slices, various studies determining the center of rotation

(COR) were conducted by three types of approaches: using pairs of projection images, the relative offset function of the rotation axis, or the center of mass[7,8,9,10,].

Afterwards, Jun and Yoon[11] provided a solution to obtain an ideally aligned reconstruction on a projection image set based on data obtained from the X-ray microtomography beamline at the National Synchrotron Light Source. The critical part was that when movement of the specimen occurred during scanning or when tilt error of the rotation axis occurred by rotating the axis with a slope during acquisition of the projection image set by scanning, it was impossible to obtain an ideally aligned reconstruction of the specimen by simply adjusting the COR in the sinogram or projection image set. To solve this problem, we developed a method for a rigid specimen to obtain a reconstructed volume in virtual space satisfying the Helgason-Ludwig consistency condition (HLCC), which essentially characterizes the range of the Radon transform. Since then, Jun and Kim[12] found a theory for elastic specimens with specific motion in computed tomography using parallel beams. The method presented above transforms a specimen with certain types of elastic motion, specifically, periodic motion, a regular expansion or contraction, and an elliptical expansion or contraction, into a rigid specimen of the desired size to obtain ideally aligned reconstructions of a rigid specimen in virtual space using the HLCC for a rigid specimen. In addition, there have been a few other studies on 3D visualization methods for elastic specimens using ultrasound or CT video[13,14,15,].

In this paper, we introduce the virtual multi-alignment method (VMAM) to transform a projection image set of a combined elastic sample to a rigid projection image set, which can be applied to living cells or animal organs. This method obtains an ideally multi-aligned reconstructed volume of a rigid specimen using the necessary number of projection images for an elastic specimen. We expect the VMAM to be applied to actual elastic samples to obtain an ideally multi-aligned reconstructed volume.

Image sample acquisition

Here, we reconstruct one slice using an actual projection image set and the virtual alignment method (VAM), which is called the virtual focusing method[11,12]. This projection image set consists of 1200 projection images obtained at the microtomography beamline with a 15 keV energy for approximately 4 hours. Since the energy of the beamline affects the specimen, the incident intensity on the specimen, or flat field images, is measured every 60 projection images. Because we cannot measure the correct incident intensity of the specimen for all

projection angles, a slight error will occur. Therefore, this results in a complex internal structure in the reconstructed slice.

The projection image set related to Fig. 1 does not have translation nor tilt errors of rotation axis and specimen. This example is used to show how the COR is determined from the sinogram. We found a projection image set with two high-density areas at one axial level, which can also serve as fiducial markers[16,17,18,19,20,21]. Each center point of those high-density areas can play a role as a reference point (for details, see Fig. S1).

Fig. 1a is obtained at a projection angle of $30°$ and has two high-density areas at the axial slice at the level 460 of the projection image, which is marked with a red arrow. Because all center points of high-density areas in the projection image set satisfy the HLCC, this projection image set does not have any translation nor tilt errors so that the axial level can play a role of a common layer. Because it becomes harder to distinguish the trajectory of the 2nd high-density area when shrinking the sinogram in Fig. 1b to 845×669 pixels, from the actual size of 1340×1200 pixels, we slightly increase the density of this area to make it look clear. Here, each pixel has a size of $4\ \mu m$.

As the position for the 1st fixed point in the sinogram (Fig. 1b) has a minimum at a projection angle of $156°$, the real COR needs to meet the trajectory of the 1st fixed point at $66°$. Similarly, the position for the 2nd fixed point has a minimum at $172°$, and hence the real COR should meet the trajectory of the 2nd fixed point at $82°$. To apply a filtered inverse Radon transform, the COR should be centered in the sinogram. Since this sinogram can have an ideal sinogram pattern (ISP) by positioning the real COR in the center of the image, one needs to either insert 17 rows below or delete 17 rows above to have the real COR in the center of Fig. 1c. The ISP is defined by a sinogram pattern for the axial level of the projection image set without translation and / or tilt errors. Here, we inserted the 17 rows below. The reconstructed slice of Fig. 1d is obtained from the sinogram in Fig. 1c. The positions of the high mass attenuation coefficient[22] (MAC) areas corresponding to the two high-density areas marked in Fig. 1a are indicated by pink and blue arrows in Fig. 1d, respectively. There is also a relation between the position of each fixed point of the reconstructed slice and the trajectory function $T_{r,\varphi}(\theta)$, which is indicated with pink and blue arrows in the sinogram of Fig. 1b, and is given in the following:

$$T_{r_1,\varphi_1}(\theta) = r_1 \cos(\theta - \varphi_1),$$

$$T_{r_2,\varphi_2}(\theta) = r_2 \cos(\theta - \varphi_2),$$

where r is the distance between the rotation axis and the fixed point p, θ is the projection angle, and φ is the angle between the line $\overleftrightarrow{Op}$ and the line orthogonal to the X-ray direction. In Fig. 1e, the left image shows a

region of interest of 600×600 pixels extracted from the reconstructed slice of Fig. 1d. The middle image shows the segmentation[23] of the same area.

Elastic-type multisection system

In this section, we introduce a method to obtain an ideally aligned reconstruction for each pixel of an elastic specimen by using image samples with an internal pattern arising from ring artifacts at a low intensity (see right panel in Fig. 1e). For the sinogram of the image samples with the ISP, we still need to observe the internal pattern of the soil grains resulting from the ring artifacts in the reconstructed slice obtained by the filtered back projection. The new method introduced here is to obtain the ideally multi-aligned reconstruction by converting the size of a specimen having various elastic motions to a desired size through a mathematical transformation in the sinogram. In fact, this alignment method for elastic specimens can rearrange the projection image set so that it meets the HLCC for a rigid specimen, regardless of size, as long as the size of the specimen changes randomly and can be measured. To examine the core part of the VMAM, we make the following two assumptions:

1) In the projection image set, the common layer corresponding to a certain height of a specimen is selected.

2) Although the size of the object changes, the total X-ray MAC for the pixels is identical.

If each section contracts at a constant rate and if we have the shape of the specimen for both $0°$ and $179.85°$, then we can predict the size and type of the object corresponding to each angle in the sinogram. In addition, for an object contracting continuously, we can obtain the contraction coefficient in the direction perpendicular to the beam direction by comparing its projected sizes corresponding to $0°$ and $179.85°$. We created a new projection image set consisting of 1200 images, based on the previous X-ray microtomography projection image set. Fig. 2a shows the structure of the specimen at each angle; $0°$, $89.85°$, and $179.85°$ (top left, top right and bottom left, respectively). Here, the left part is the elliptic section with elliptic motion[13], and the right part of the image specimen is the regular section with regular motion in which the size is expanded or contracted while the shape is maintained. Specifically, whenever the projection angle varies, the elliptic section contracts by $0.054\% \times 0.036\%$, and the regular section contracts overall by $0.054\% \times 0.054\%$. The right side of Fig. 2b is related to consistent changes in the projection angle of $0.15°$, but the left side is related to irregular change. We create a sinogram at consistent intervals so that the change of the projection angles can be a constant $0.15°$

when the sinogram for the elliptic section is converted into a rigid-type sinogram. In the rigid-type sinogram, the VAM only allows the non-ISP to be converted to the ISP locally for the section, which is called a locally aligned pattern. When a specimen can be divided by several sections based on their motions, we refer to the VAM applied to each section as the VMAM. In the case of a 3D specimen, the LAP is defined within a common layer of each section. Since there is only a 50% difference between the contraction rates for the major and minor axes[12], it is possible to produce good results only with the sinogram on the left of Fig. 2b. However, we use two sinograms to obtain mathematically better results for the elastic motion that changes significantly in one direction. Each sinogram in Fig. 2c has the LAP for each section by transforming elastic motion to rigid motion[13] and by using the VAM[12] for rigid specimens. Each slice in Fig. 2d was reconstructed from each sinogram of Fig. 2c, and a combined reconstructed slice (Fig. 2e) was obtained using the region of interest of each section (see Fig. S2 and Fig. S3 for ideally multi-aligned reconstructions of other image samples from TomoBank[24]).

Result and Discussion

The size of the projection image used in Fig. 1 is 500×1340 pixels. The trajectory indicated by the blue arrow in Fig. 1b was slightly emphasized by increasing the density of the high-density area as it appeared dimmed during the sinogram reduction. Fig. 1d is a reconstructed slice using a real sinogram without any change of densities. Here, we obtained a reconstructed slice as an image sample containing two high-MAC areas, but we found that the position of each high-density area becomes more blurred in the sinogram of Fig. 2b. In other words, when we compare the actual sinogram of Fig. 1b obtained by X-ray tomography acquisition with the sinogram of Fig. 2b obtained by applying the Radon transform to the segmented reconstruction in Fig. 1e, we found that the trajectory in Fig. 2b was more blurred than the trajectory in Fig. 1b, even though we gave the same density increments to the 2nd high-density area for each sinogram in Fig. 1b and Fig. 2b. To examine the blurring phenomena, we placed 109 pixels with a high MAC at the center and edge of the sample in a reconstructed slice (Fig. 3a). Here, we intentionally used an image sample without a high-MAC area. The purpose of this observation is to investigate the changes in the number of pixels with a high MAC in the reconstructed slice when we apply a pair of the Radon transform and the filtered inverse Radon transform repeatedly under ideal conditions such that the thickness of the object and the pixel value in the projection image are directly proportional for the Radon transform, and a well-aligned projection image set is given for the

filtered inverse Radon transform. Because we expected a reduced MAC for areas after reconstruction of the reconstructed slice with a high-MAC area, we started to observe an image sample without a high-MAC area at the beginning and then added the high-MAC area after reconstruction. Then, we compared the number of voxels (Fig. 3b) maintaining the intensity over 80% and the number of projection images in the projection image set using the filtered inverse Radon transform. In addition, we investigated the number of voxels that maintain an intensity of 80% or more when using the Radon transform and the filtered inverse Radon transform repeatedly. As shown in Fig. 3c, the high-MAC area decreases rapidly when the filtered inverse Radon transform is used twice; therefore, we emphasize the 2nd high-density area of the image sample when creating an elastic-type sinogram.

When we have a projection image set of several elastic motions without information about the specimen, we need at least four fixed points on the common layer for each elastic motion to obtain a projection image set of rigid motion. In fact, in the case of regular elastic motion in one common layer, it is sufficient to have two fixed points on one straight line passing through the center of the contraction and expansion to calculate the exact contraction and the expansion coefficient. For elliptical elastic motion, it is sufficient to have two fixed points on both the major and minor axes. Principally, a specimen made of one material will have various expansion coefficients depending on the thickness of each part even if the same external force is provided, which means that one specimen can be divided by several sections. If one could accurately calculate the motions of an object, one could theoretically obtain an ideally aligned reconstruction by the VAM. However, since the number of fixed points in a projection image set is generally insufficient, it is difficult to distinguish the motion of an object with a limited number of fixed points in the projection image set. If the specimen contracts or expands in the direction of the axial level, it will be difficult to obtain a well-aligned reconstructed volume, because as many common layers are required for each section as the number of dimensions divided by voxel size. In this paper, to simplify the problem, we used a specimen with two sections having different elastic motions that continuously contract in the common layer. Moreover, we assumed that we know the elastic motion for each section.

If we can measure the initial size and the last size of the continuously varying section and obtain each projection image at a constant time interval during scanning, each elastic-type projection image can be converted into a rigid-type projection image through the measured changes. If there is one fixed point in each section, it is possible to obtain an ideally aligned reconstructed volume for the section through the VAM. Furthermore, suppose that you have two objects with the same period of motion. For example, the cardiac cycle is closely

related to the pulse cycles. If the time interval between each cardiac projection image is constant and the time is a multiple of the pulse cycles, then the elastic-type heart will act as a rigid specimen in the projection image set. Although the cycle is not constant, as long as the shape of the heart can be predicted through the pulse, it would be possible to obtain a projection image converted from elastic motions to rigid motions. Of course, it is best to obtain a rigid-type projection image set through measuring the cardiac cycle precisely. It is true that there are still many open problems; however, we believe that the VMAM is a critical tool to find a general solution for specimens with elastic motion.

Method

In parallel-beam tomography, the VAM was introduced to obtain an ideally aligned reconstruction of a rigid specimen. (Jun and Yoon, 2017). If we have a fixed point in a common layer to move along the sinusoidal curve $T_{r,\varphi}$ with respect to a virtual COR $T_{0,\varphi}$, all the points of the specimen in a projection image (even with axial level errors or translation errors of the rotation axis or specimen) will satisfy the HLCC. Moreover, if there exists an additional vertical tilt error of the rotation axis, then we need two fixed points to achieve the ISP.

A method of creating a sinogram with the ISP was then developed using a projection image set of the elastic specimen with a specific motion (Jun and Kim, 2018). This method shows how to convert an elastic-type projection image set into a rigid-type projection image set to satisfy the HLCC based on a common layer that contains a common part of the rigid specimen in the reconstructed space.

Here, we show how to convert a combined elastic specimen to a rigid specimen using the VMAM and a method of transforming a specific elastic specimen to a rigid specimen. Fig. 4 shows how to obtain each ideally aligned reconstruction when two different elastic sections are combined in the common layer, which is the core technology of the VMAM, as explained below.

Step 1: Convert each elastic sinogram to a rigid sinogram.

Step 2: In each rigid sinogram, transform the non-ISP to the LAP using the VAM.

Step 3: Construct an ideally aligned reconstruction using each sinogram with the LAP.

Step 4: Create a combined rigid reconstructed slice using the region of interest of each section.

References

1. Larabell, C.A. & Nugent, K.A., Imaging cellular architecture with X-rays, Curr Opin Struct Biol.20, 623-631 (2010).
2. Parkinson, Y. D., Knoechel, C., Yang, C., Larabell, C. A., & Le Gros, M.A. Automatic alignment and reconstruction of images for soft X-ray tomography. *J. Struct. Biol.***177**, 259–266 (2012).
3. Lam, Y.M., Chen, X., Marean, C.W. & Frey, C.J., Bone density and long bone representation in archaeological faunas: comparing results from CT and photon densitometry, J. Archaeol.Sci. 25,559-570 (1998).
4. Zhu, W., Gaetani, G.A., Fusseis, F., Montesi, L. G. J. & De Carlo, F. Microtomography of partially molten rocks: three-dimensional melt distribution in mantle peridotite, Science, 332, 88-91, (2011).
5. Fu, Q., Saiz, E. & Tomsia, A. P., Bioinspired Strong and Highly Porous Glass Scaffolds, Adv. Funct. Mater. 21, 1058-1063 (2011).
6. Bautz, W. & Kalender, W. Radiologe, 45, 350355 (2005).
7. Pan, Y., De Carlo, F. & Xiao, X. Automatic detection of rotational centers using GPU from projection data for micro-tomography in synchrotron radiation. *Proc. SPIE*, **8313**, 831328 (2012).
8. Gürsoy, D., De Carlo, F., Xiao, X. & Jacobsen, C. TomoPy: a framework for the analysis of synchrotron tomographic data. *J. Synchrotron Rad.* **21**, 1188–1193 (2014).
9. Donath, T., Beckmann, F. & Schreyer, A. Automated determination of the center of rotation in tomography data. *J. Opt. Soc. Am. A*, **23**, 1048–1057 (2006).
10. Yang, Y. et. al. Registration of the rotation axis in X-ray tomography. *J. Synchrotron Rad*., **22**, 452-457 (2015).
11. Jun, K. and Yoon, S. Alignment Solution for CT Image Reconstruction using Fixed Point and Virtual Rotation Axis. *Sci. Rep***.** **7**, 41218; doi: 10.1038/srep41218 (2017).
12. Jun, K. and Kim, D. Alignment theory of parallel-beam computed tomography image reconstruction for elastic-type objects using virtual focusing method. *Plos One,* **13(6)**:e0198259 (2018)
13. Lu, M., Wu, D., Lin, W.H., Li, W., Zhang, H., Huang, W. A stochastic filtering approach to recover strain images from quasi-static ultrasound elastography. *Biomed Eng Online*.,**13**:15 (2014).
14. Gao, Z. et al. Robust estimation of carotid artery wall motion using the elasticity-based state-space approach. *Med Image Anal*.,**37**:1-21 (2017).
15. Walker, S.M. et al. In vivo time-resolved microtomography reveals the mechanics of the blowfly fight motor. *Plos Biol.* **12**,e1001823 (2014).

16. Kremer, J.R., Mastronarde, D.N. & McIntosh, J.R. Computer visualization of three-dimensional image data using IMOD. *J Struct Biol.*,**116**:71-76 (1996).
17. Sorzano, C.O.S. et al. XMIPP: a new generation of an open-source image processing package for electron microscopy. *J Struct Biol*.,**148**:194-204 (2004).
18. Amat, F. et al. Markov random field based automatic image alignment for electrontomography. *J Struct Biol.*,**161**:260-275 (2008).
19. Chen, H., Hughes, D.D., Chan, T.A., Sedat, J.W. & Agard, D.A. IVE (Image Visualization Environment): a software platform for all three-dimensional microscopy applications. *J Struct Biol*.,**116**:56–60 (1996).
20. Liu, Y., Penczek, P.A., Mcewen, B.F. & Frank, J. A marker-free alignment method for electron tomography. *Ultramicroscopy*.,**58**:393–402 (1995).
21. Zheng, S.Q. et al. UCSF tomography: an integrated software suite for real-time electron microscopic tomographic data collection, alignment, and reconstruction. *J. Struct. Biol*.,**157**:138-147 (2007).
22. Hubbell, J.H. Photon mass attenuation and energy-absorption coefficients. *Int. J. Appl. Radiat. Isot.* **33**, 1269-1290 (1982).
23. Oh, W. and Lindquist, W.B. Image Thresholding by Indicator Kriging, *IEEE Trans. Pattern Anal. Mach. Intell.* 21, 590-602 (1999).
24. De Carlo, F., et al. Tomobank: a tomographic data repository for computational x-ray science. *Measurement Science and Technology*. http://stacks.iop.org/0957-0233/29/i=3/a=034004 (2018).

Acknowledgement

The authors would like to express their gratitude to Dr. Keith Jones, a senior physicist at the Brookhaven National Laboratory, for provision of the soil projection sets.

We greatly appreciate the two reviewers and the Editorial Board Member for their helpful comments.

Author contributions Statement

K. J conceived and designed the theoretical experiments and performed the experiment.

The theoretical solutions were developed by all authors, and all authors wrote the paper.

Competing financial interests

The authors declare no competing financial and non-financial interests.

Figure Legends

Figure 1. Image sample acquisition process. Experimental data to observe the dissolution of the grains of Hanford soil in a polyether ether ketone column from the X-ray microtomography beamline at the National Synchrotron Light Source. The projection image set has a projection angle variation of $0.15°$ and consists of 1200 projection images. (a) Projection image with 500×1340 pixels at a projection angle of $30°$. (b) COR, the projected rotation axis of the stage, determination when a sinogram has two fixed points. The white vertically long rectangle sitting at a projection angle of $30°$ corresponds to the yellow horizontally long rectangle in Fig. 1a. Each sinusoidal curve marked by the pink and blue arrows has a minimum at projection angles of $156°$ and $172°$, respectively. (c) Set the COR as the center of the sinogram to apply the filtered inverse Radon transform by inserting 17 rows at the bottom of Fig. 1b to have the COR in the center of Fig. 1c. (d) Analysis of the corresponding positions of two high-MAC areas in the reconstructed slice to two high-density parts of (b). (e) Part of the reconstructed slice image, particularly containing soil grains with a size of 600×600 pixels (left), an image corresponding to the region of interest by using segmentation of the area of the soil grains (middle), and an enlarged image to see the internal pattern of the soil grains (right).

Figure 2. Virtual multi-alignment method for an elastic specimen combined by two different elastic sections. When two different types of elastic sections are contracted during scanning, mathematical modifications cannot produce an ideal sinogram pattern as a whole. (a) The process of deformation of two elastic sections during scanning. The structure of the image specimen was captured at different projection angles ($0°$, $89.85°$, $179.85°$; top left, right and bottom left, respectively). In the bottom right panel, the elliptical section contracts with reduction rates of 0.036% in the horizontal direction and 0.054% in the vertical direction, and the regular section contracts with a reduction rate of 0.054%. (b) Sinograms in the process of

deformation. The amplitude of the sinogram gradually decreases with respect to the projection angle. After converting the left sinogram to a rigid-type sinogram, the change in the projection angle will become constant $0.15°$. In the right sinogram, the projection angle consistently changed by $0.15°$ based on the X-ray microtomography beamline at the National Synchrotron Light Source. (c) For each section, we used the VAM and rigid-type conversion to satisfy the ideal sinogram pattern. (d) Locally aligned reconstructions from the sinograms of (c) the elliptic section (left) and regular section (right). (e) Multi-aligned reconstruction combining the locally aligned reconstructed sections from (d) using the region of interest of each section.

Figure 3. Spread of the high-MAC area. The pixel number of the high-MAC area is reduced by spreading the MAC whenever the filtered inverse Radon transformation is applied under ideal conditions such that the thickness of the object and the pixel value in the projection image are directly proportional for the Radon transform, and a well-aligned projection image set is given for the filtered inverse Radon transform. Because we expect a reduced MAC for areas after reconstruction of the reconstructed slice already having a high-MAC area, we started to observe this for an image sample without a high-MAC area at the beginning and then added the high-MAC area after reconstruction. (a) The test object has two high-MAC areas of 109 pixels for each. (b) The number of pixels maintaining a high-MAC area of 80% or more of the initial MAC in a reconstructed slice according to the projection image. (c) Reduction of the high-MAC pixels when using the Radon transform and the filtered inverse Radon transform.

Figure 4. Flowchart of the process of obtaining a 3D reconstructed volume of the elastic sections using the virtual multi-alignment method. To obtain an ideally multi-aligned volume as a whole, it must be divided into sections according to the type of elastic motion. We then calculate the thickness of the common layer, taking into account the amount of change in each section in the axial direction to create an elastic-type sinogram. After changing the elastic-type sinogram to a rigid-type sinogram, we change the non-ISP to the LAP using the VAM. We use the LAP to achieve the ideally aligned reconstruction. Then, for each common layer, all reconstructed slices are combined to obtain a reconstructed volume for one section. We obtain the ideally multi-aligned volume as a whole by repeating the operation of a section for all sections and adding them together using the region of interest of each section.
