# Supplementary material for: Virtual multi-alignment theory of parallel-beam CT image reconstruction for elastic objects
Source: Sci Rep. 2019 May 2;9:6847. doi: 10.1038/s41598-019-43331-2 (PMC6497627; doi:10.1038/s41598-019-43331-2)
Supplement: Supplementary file 1 — All supplementary files [file 41598_2019_43331_MOESM1_ESM.zip › Marked_Supplementary.pdf]

Virtual multi-alignment theory of parallel-beam CT image reconstruction for elastic objects

Kyungtaek Jun[a,*], Joeun Jung[b]

[a]IM Technology Research Center, 6, Teheran-ro 52-gil, Gangnam-gu, Seoul, 06211, Republic of Korea

[b]School of Mathematics, Korea Institute for Advanced Study, 85 Hoegiro, Dongdaemungu, Seoul, 02455, Republic of Korea

*ktfriends@gmail.com

Supplementary Figures (S1-S3)

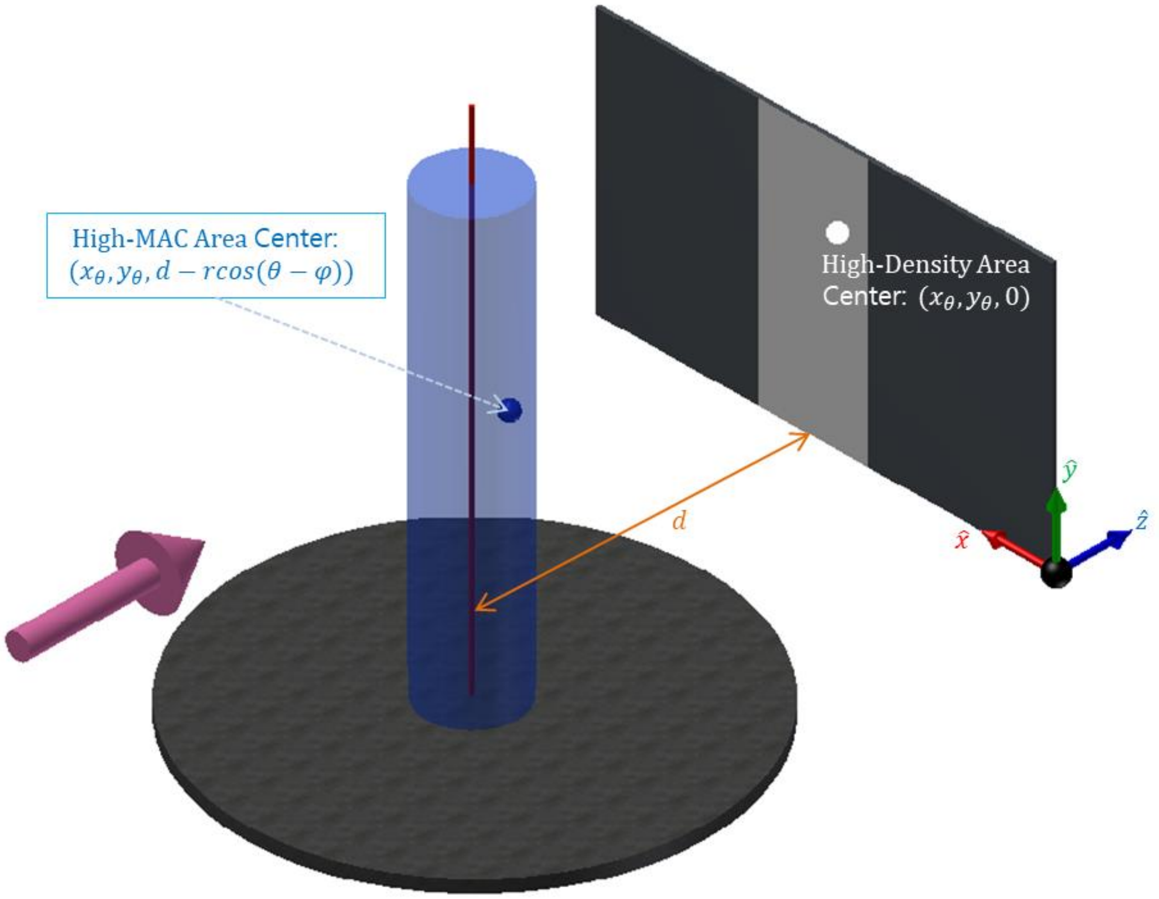

Figure S1. Fixed point acquisition. Let a specimen have a small size of X-ray high-MAC area inside it and rotate during scanning on the stage as shown in the figure. When the distance between the rotation axis and the CCD is equal to d, if for a given projection angle θ, the projection of the center point $(x_\theta, y_\theta, d - rcos(\theta - \varphi))$ of the high-MAC[22] area of the specimen can be obtained by the center point $(x_\theta, y_\theta, 0)$ of the high-density area in the CCD plane, then we call the center point (x_θ, y_θ) on the CCD as the fixed point. Here, r is the distance between the center point of high-MAC area and the rotation axis, and φ is the angle between the vector $\vec{r}$ from the rotation axis and the line orthogonal to the X-ray direction. The fixed point should be computable or distinguishable throughout the projected image set. We can rearrange the projection image set by moving the fixed point using the VAM. Generally, the center of the high-MAC area of the specimen can be calculated well. For instance, a point having a particular shape, such as the cusp of an object, is distinguishable throughout the projection image set; hence, the point can be used as a fixed point. Moreover, we can calculate the center of attenuation for the desired part and use it as a fixed point under ideal conditions: either if the thickness and energy absorption of the object are linear and the the projected object exists within the CCD, or if the thickness and energy absorption of the object are linear, and a common layer set exists and contains the same part of the object in the projection image set.

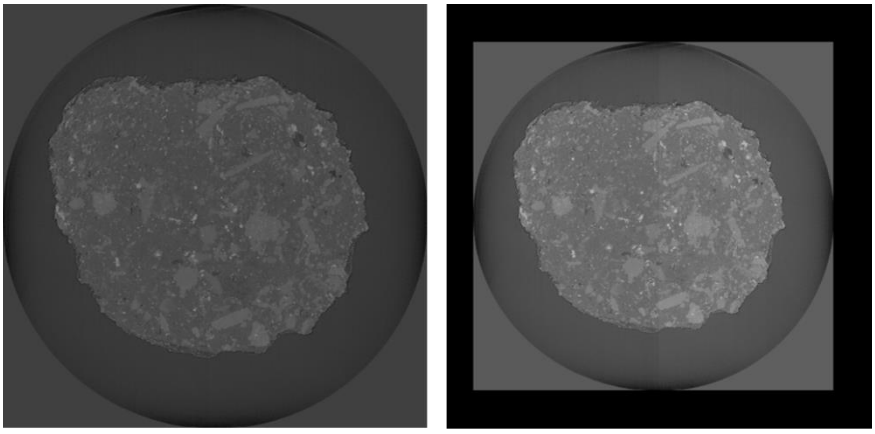

a

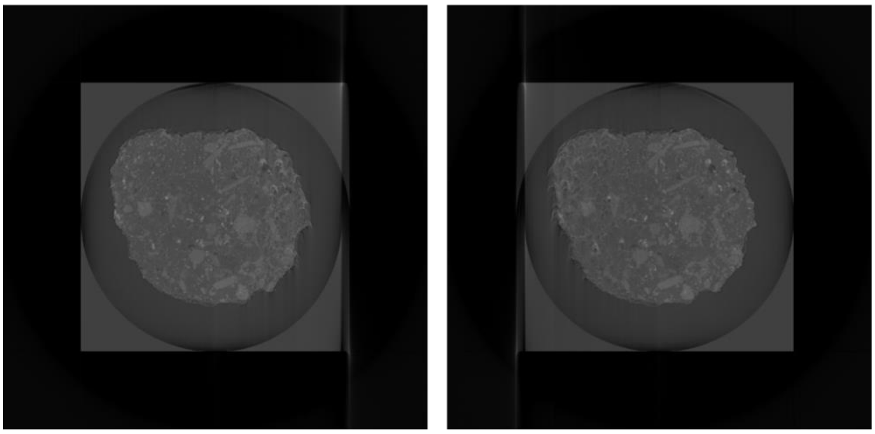

b

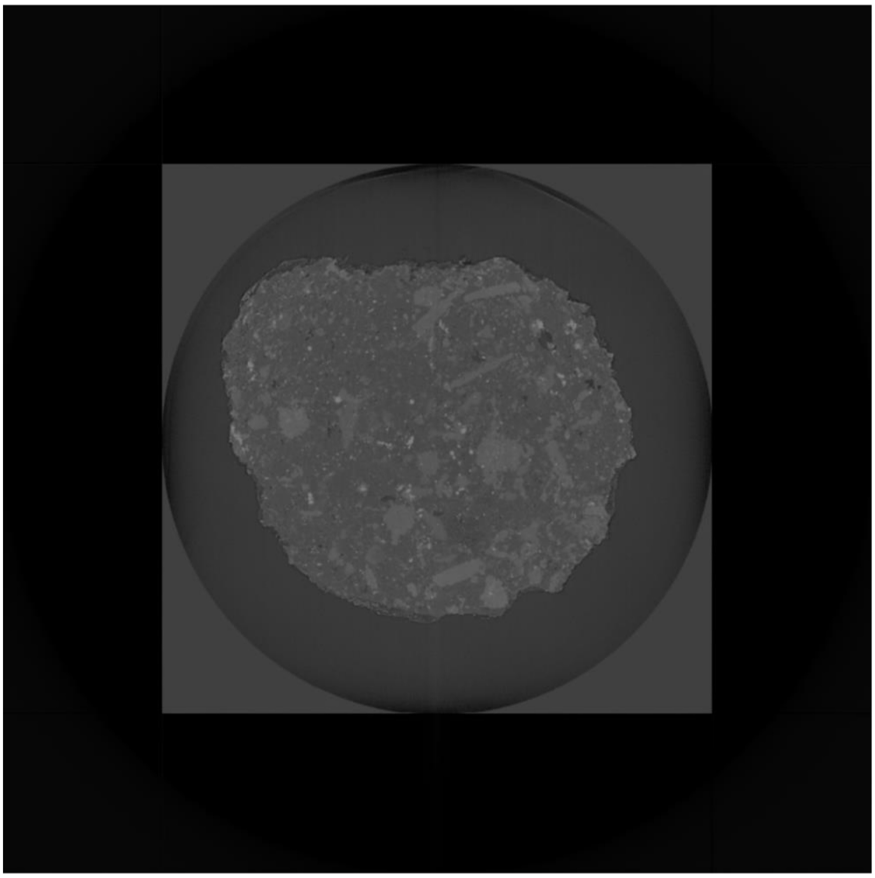

c

Figure S2. Ideally multi-aligned reconstruction of an image sample from TomoBank with the same two elastic motions as in Fig. 2 using the VMAM. (a) Image samples used at projection angles of 90 ° and 179.85 °. (b) Locally aligned reconstructions for each section. (c) Multi-aligned reconstruction combining the locally aligned reconstructed sections.

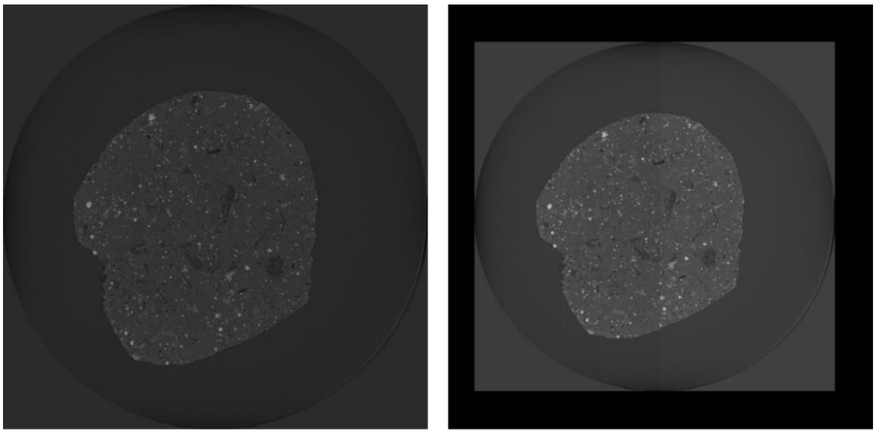

a

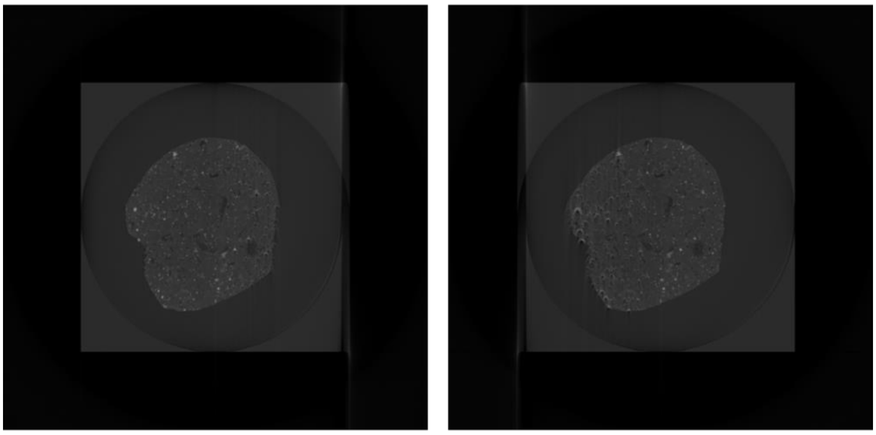

b

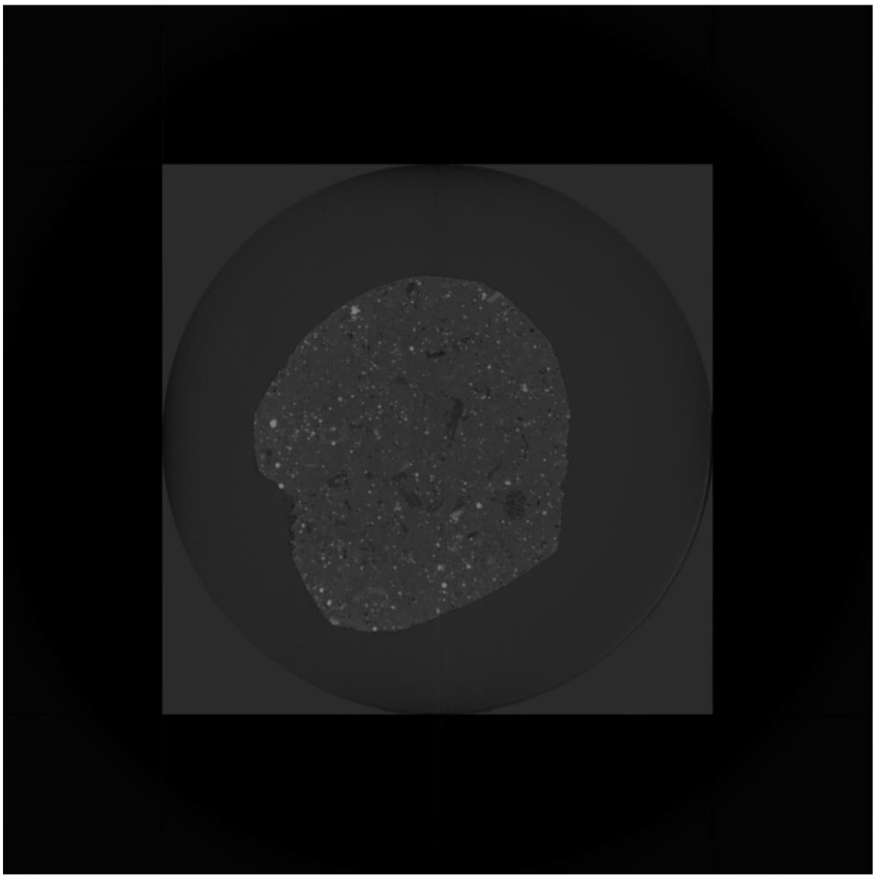

c

Figure S3. Ideally multi-aligned reconstruction of an image sample from TomoBank with the same two elastic motions as in Fig. 2 using the VMAM. (a) Image samples used at projection angles of 90 ° and 179.85 °. (b) Locally aligned reconstructions for each section. (c) Multi-aligned reconstruction combining the locally aligned reconstructed sections.

Supplementary Video (S1)

Supplementary Video S1. Changes in the sample shown in Fig. 2 during scanning. This video consists of 330×330 pixels and has 80 frames per second.
